# A Review of Fast Bubble-Driven Micromotors Powered by Biocompatible Fuel: Low-Concentration Fuel, Bioactive Fluid and Enzyme

**DOI:** 10.3390/mi9100537

**Published:** 2018-10-22

**Authors:** Qingjia Chi, Zhen Wang, Feifei Tian, Ji’an You, Shuang Xu

**Affiliations:** 1 Hubei Key Laboratory of Theory and Application of Advanced Materials Mechanics, Department of Mechanics and Engineering Structure, Wuhan University of Technology, Wuhan 430070, China; qingjia@whut.edu.cn (Q.C.); wangzhen@whut.edu.cn (Z.W.); 2 School of Life Science and Engineering, Southwest Jiaotong University, Chengdu 611756, China; tulwar@home.swjtu.edu.cn; 3 School of Energy and Power Engineering, Wuhan University of Technology, Wuhan 430063, China; 256741@whut.edu.cn

**Keywords:** bubble-driven micromotors, biocompatible, low-concentration fuel, enzyme

## Abstract

Micromotors are extensively applied in various fields, including cell separation, drug delivery and environmental protection. Micromotors with high speed and good biocompatibility are highly desirable. Bubble-driven micromotors, propelled by the recoil effect of bubbles ejection, show good performance of motility. The toxicity of concentrated hydrogen peroxide hampers their practical applications in many fields, especially biomedical ones. In this paper, the latest progress was reviewed in terms of constructing fast, bubble-driven micromotors which use biocompatible fuels, including low-concentration fuels, bioactive fluids, and enzymes. The geometry of spherical and tubular micromotors could be optimized to acquire good motility using a low-concentration fuel. Moreover, magnesium- and aluminum-incorporated micromotors move rapidly in water if the passivation layer is cleared in the reaction process. Metal micromotors demonstrate perfect motility in native acid without any external chemical fuel. Several kinds of enzymes, including catalase, glucose oxidase, and ureases were investigated to serve as an alternative to conventional catalysts. They can propel micromotors in dilute peroxide or in the absence of peroxide.

## 1. Introduction

Micromotors, a kind of micro structure, can convert chemical energy, electricity, or light energy from the surrounding environment into kinetic energy [1,2]. They display lots of advantages in practical applications, including tiny size, large thrust-to-weight ratio, and low power consumption [3]. Micromotors are extensively applied in cell manipulation [4,5], payload delivery [6,7,8,9], and in the removal of toxicant components [10,11]. For instance, micromotors have been applied in the treatment of stomach infections [12] and thrombus therapy [13]. Due to limitations of the means of driving them, many micromotors exhibit a speed of dozens of micrometers, such as bimetallic nanorods [14] and Janus nanorods [15]. In practical applications, micromotors with higher speeds and good biocompatibility are highly desirable [16,17]. Thus, bubble-driven motors have gained lots of attention due to their apparent advantages in terms of speed. They can reach fast (>10 μm/s) and even superfast speeds (100 μm/s) upon bubble ejection, thanks to an ingenious design of the motor body [11,18,19]. For example, a bubble-propelled microjet displays a superfast speed of 10,000 μm/s [20]. A micromotor of zinc as the inner wall and polyaniline as the outer wall can reach a speed of 1000 μm/s. Micromotors of Pt/Co/Ti as the inner wall can reach similar speeds [21]. Moreover, they can perform special tasks in the absence of external forces, and can be rather simple in structure. The propulsion process is almost not affected by ion concentration.

Bubble-driven micromotors move forward through detaching or bursting bubbles [22]. The bubble is generated through reactions between the motor material and the solution. Most micromotors need fuel, including H_2_O_2_, acid, alkaline, Br_2_, or I_2_ solutions to generate bubbles [19]. Among them, H_2_O_2_ is the most commonly-used fuel source. A surfactant is added to promote the release of bubbles [23]. Bubble-driven motors are commonly divided into Janus particles and tubular structures [24,25]. The motors move due to a recoil effect caused be the growth and ejection of bubbles generated by the motors. The motor was built into an asymmetrical structure to guide the bubble to discharge directionally, while the driving force points in the opposite direction. The surface of the motor can be modified by various functional ligands to make it suitable for a variety of practical applications including biomedicine, chemical industry and environmental clearance [19,26].

Bubble-driven micromotors are capable of spontaneous directional motion by symmetry breaking, which is implemented into the anisotropic composition, shape, or surface reactions [27,28,29]. For instance, the hemisphere of a Janus motor is coated with a catalyst to create an asymmetric generation of bubbles [14,30]. Due to the perfect catalytic activity in decomposing H_2_O_2_, the rare metal platinum (Pt) has been the most widely-used catalytic agent to prepare micromotors of various geometries, including tubular engines [31,32], and Janus motors [22,33,34]. Considering the scarcity and high cost of Pt, researchers also used Pt-free catalysts to propel motors. For instance, reactive micromotors have been fabricated based on the reactions of metals with water and acid [3,35]. Many in vivo applications of drug delivery of micromotors have taken advantaged the existence of acid in the stomach [12,36,37]. Moreover, enzyme-propelled motors are also proposed as a new strategy due to the good catalytic performance and native biocompatibility of enzymes [12,38].

As for Janus particles, small oxygen bubbles are formed on the catalytic surface, and they continue to grow under the supply of surrounding dissolved oxygen molecules. The detachment acts as a net momentum on the motor, and induces a driving force and an initial horizontal velocity on the motor opposite to the catalytic surface [14]. The geometrical construction of the conical motor is asymmetric in nature. The bubble moves spontaneously towards the larger open side under the action of a pressure difference [35]. Bubbles in cylindrical motors do not have to move towards a specific side at the beginning. They choose the initial exit opening randomly and move towards the direction continually. As long as peroxide is present, a new bubble would be generated and released after the detachment of the last bubble. Thus, continuous detachment events of the bubbles drive the motor ahead persistently.

Fuel concentration is a critical determinant of motor motility. The velocity of the bubble-driven motor is positively correlated with the fuel concentration, which was validated by a large number of reports [21,39,40]. Thus, lots of researchers still rely on high fuel concentrations to promote motion performance. Reviews have variously focused on the physical strategies [41], fabrication techniques [42], and specific applications [36,43,44]. Additionally, several publications about the moving speeds of bubble-driven micromotors have been reported [16,45]; however, few discuss the motion performance with biocompatible fuels.

Our previous studies have underlined the important role of fuel concentrations in motor locomotion [45,46]. The toxicity associated with concentrated fuel has hindered practical applications of micromotors, especially in the biomedical area [43,47]. Bubble-driven micromotors with high speeds and good biocompatibility are highly desirable [16]. In recent years, the authors have constructed delicately-designed micromotors with good motility and biocompatibility, which are fueled by a low-concentration fuel, water, and enzymes.

## 2. Low Concentration of Peroxide

### 2.1. Janus Micromotors

**Figure 1:**
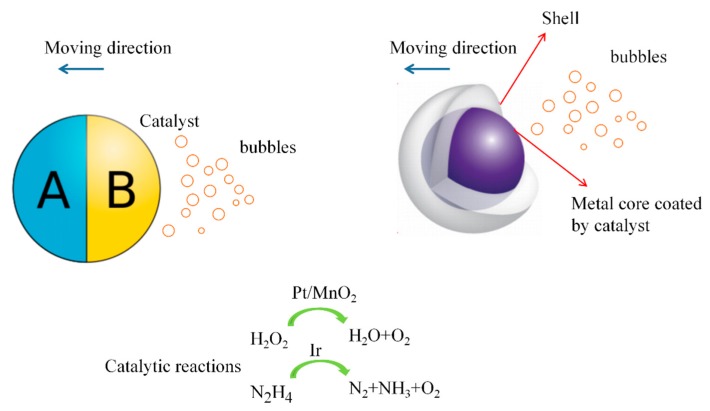
A Janus micromotor is designed by half-coating catalyst onto one side or coating catalyst onto the metal core of a nanoshell motor. Catalytic reactions produce gas to bring the motor forward. Small oxygen bubbles are formed, and they continue to grow under the supply of dissolved oxygen molecules. The detachment of bubbles acts as a net momentum on the motor, and induces a propulsion force and an initial velocity towards the opposite side.

A commonly-seen example of a bubble-driven Janus micromotor involves the use of colloid particles half-coated by a catalyst, as shown in Figure 1. The directional movement is due to the catalytic reaction on the catalytic surface, generating oxygen or hydrogen to provide a driving force [48]. The speed of Janus micromotors is dependent on fuel concentration, particle size, and thickness of the catalyst [49,50]. Due to limitations of size and propulsion manner, small colloid particles typically exhibit low speeds, even at very high fuel concentrations. For example, motors with a diameter of 1.62 μm display a speed of 3 μm/s at 10% peroxide [51]. Pt-coated colloidal particles with a diameter of 2 μm/s exert a speed of several micrometers per second in a wide range of H_2_O_2_ [3]. Polystyrene-Pt colloid particles with a diameter of 2 μm and silica-Pt colloids exhibit a speed of 9 μm/s and 6 μm/s [22,49]. Small carbonate-based Janus particles move at a speed of 1.8 μm/s in acidic solution generated by HeLa cells [52].

A precise procedure is followed in order to enhance the speed of the Janus particles. The speed of the directional motion can be enhanced a few times by selectively slowing down the rotational diffusion [53]. Particles of about 2 mm diameter are observed to move at a speed of 16 μm/s. Janus particles propel away from the catalytic site, which is consistent with the nanobubble detachment mechanism [54]. Experimental observations showed a very large Janus micromotor with an average diameter of ~45 μm exerts an instantaneous velocity of 14 cm/s in 5% H_2_O_2_ [33]. In another study, microporous large carbon Janus micromotors show a very fast speed of 190 μm/s in 2% peroxide [55]. The rapid movement of the micromotor, together with corresponding fluid mixing, made the motor a highly-efficient platform in water purification. Very large colloid size (~60 μm), small mass, together with reduced fluid drag forces contribute to the fast speed of the micromotor. There are also Pt-free micromotors which were designed on silver and manganese dioxide surfaces [56]. Manganese dioxide (MnO_2_) acts as another catalyst that can decompose H_2_O_2_, and is more accessible than expensive Pt. The motor could move at a speed of more than 50 μm/s in a dilute fuel, and a speed of ~25 μm/s was observed in a very low peroxide concentration of 0.1%. Taking advantage of the reactions between the iridium hemisphere layer and hydrazine solution, Gao et al. prepared micromotors with a high speed of 20 μm/s in a 0.001% hydrazine solution [57]. Hydrazine is a well-known fuel commonly used as a rocket propellant. Hydrazine can be catalyzed by iridium to produce hydrogen and nitrogen. The motor could be powered by a remarkably low concentration of 0.0000001%.

A precise design of geometric asymmetry of Janus motors could enable fast movement of the motor. Nanoshell motors with a catalyst-coated opening show significantly faster speeds than solid, spherical Janus motors. The motors display a maximum speed of 300 μm/s, while the average speed is 100 μm/s [58]. A micromotor with “coconut” structure, namely a hollow semisphere shell, was able to reach a speed of hundreds of micrometers per second upon bubble ejection in dilute fuel [30]. The speed of the micromotor was comparable to that of a phoretic micromotor with similar catalyst distribution [59]. By changing the geometry of Janus micromotors into multilayer hollow capsules, the authors prepared a new kind of bubble-driven micromotor [60]. The Janus capsule motor could move at a maximum speed of more than 1 mm/s in concentrated fuel, and ~150 μm/s in dilute solution.

### 2.2. Tubular Micromotors

Bubble-driven tubular micromotors are typically propelled by chemical reactions which occur in the inner chamber of a hollow structure. As for tubular micromotors placed in H_2_O_2_ solution, a catalyst like Pt should be embedded in the inner wall of the tube to produce bubbles. The gas molecules could nucleate, then mature into bubbles, diffuse in the tube chamber, and finally, eject from one opening of the micromotor [32]. Tubular micromotors typically show good motility performance due to their unique morphology [42]. The tube body begins to move in the opposite direction after the bursting of the bubbles. In the case of cylindrical structures, both opening ends could act as the nozzle of bubble ejection. As for the case of the conical structure, the larger opening act as the ejection nozzle. Cylindrical micromotors are more inclined to move in a straightforward manner. Using common cylindrical microtubular engines, the authors were able to obtain a speed of more than 400 μm/s at a H_2_O_2_ concentration of 5% [21]. Cylindrical motors in H_2_O_2_ solutions exhibit a speed as high as 180 μm/s [61]. Manjare et al. [32] reported that the average speeds of tubular micromotors are in the range of 100–1000 μm/s in 5% peroxide. Considering the relationship among motor velocity, motor geometry, and solution concentration, a large microjet length, small opening diameter, and large concentration is required to acquire a perfect performance of motility.

**Figure 2:**
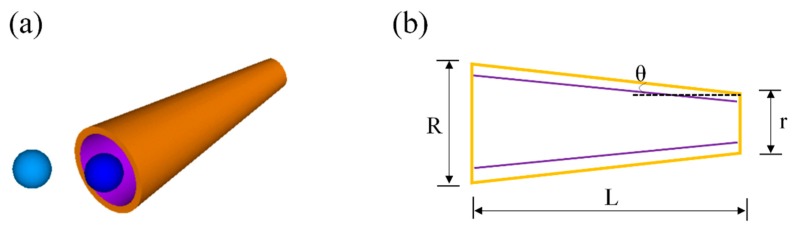
Promoting motion performance of a cone-shaped tubular micromotor by optimizing the geometry. (**a**) General schematic of a cone-shaped tubular micromotor. (**b**) A description of dimension label of conical micromotors. The motion performance of the motor could be promoted by optimizing the geometry, including reducing the ratio of length to radius or enlarging the semi-cone angle.

Moreover, tubular micromotors could acquire faster speeds by optimizing the geometry, namely, reducing the ratio of length to the radius or enlarging semi-cone angle, as demonstrated in Figure 2. Chemical reactions on the inner wall of the tube must be promoted to guarantee efficient motion in dilute fuel. Various delicate methods have been presented to promote interfacial chemical reactions. The increased solution temperature promoted the motion owing to the fact that it reduced fluid resistance and accelerated chemical reactions. It was demonstrated that the microjets acquire a superfast speed of 10 mm/s at an elevated temperature of 37 °C in 5% H_2_O_2_ solution [20]. In contrast, the microjet could completely halt at a low temperature of 2 °C. And a very low peroxide concentration of 0.25% could propel the motor to a speed of 140 μm/s. A microjet with hierarchical nanoporous walls could reach a very fast speed by enlarging the catalytic surface area and improving the reactant accessibility [62]. The authors noticed that the structure leads to a faster gas production rate and generation frequency to promote the movement; micromotors with such a sophisticated structure exhibit a 2–5-fold enhancement in speed relative to those with a smooth surface. The motor could maintain a speed of 120 μm/s in a peroxide concentration of 0.2%. A very high speed of 1077 μm/s was observed in 7% H_2_O_2_ solution. Improving catalytic reaction efficiency to promote jetting frequency could also be implemented by a combination of roll-up nanotechnology and atomic layer deposition [63]. The technique leads to an ultrafast speed of 1000 μm/s in 5% peroxide. Newly-designed tubular micromotors, prepared by modifying MnO_2_ with mixed valence iron oxide, move much faster than conventional Pt-catalyzed motors [64]. These microtubes move at a speed of 89 μm/s in a rather low fuel concentration of 0.03% H_2_O_2_.

**Figure 3:**
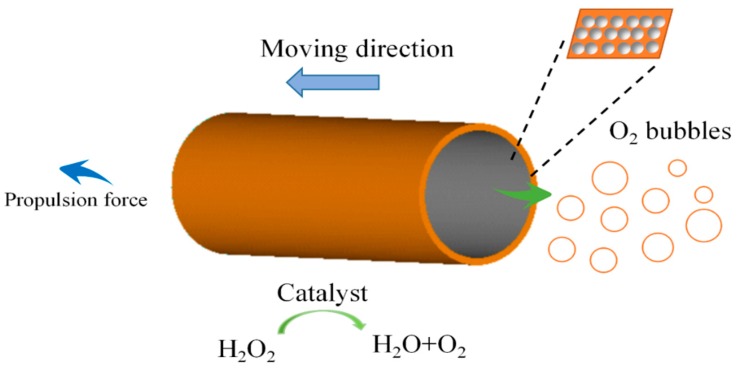
The motion of a nanojet could be promoted significantly by embedding nanoengines with Pt nanoparticles, which is called *particle-assisted rolling*. The technique fulfills this task by increasing the reaction activity from the dispersed catalytic nanoparticles. The technique is helpful for the motor locomotion with low-concentrations of fuel.

Micro-scale chemical reactions could also be fulfilled by embedding nanoparticles, or enlarging the rough surface. The method is even effective for rather tiny nanojets. Due to the size limitation, nanojets demonstrated a slower speed of 180 μm/s in very concentrated fuel [61,65]. The problem of the nanojet could be resolved by embedding nanoengines with Pt nanoparticles, which is called particle-assisted rolling [66], as shown in Figure 3. The technique fulfills this task by increasing reaction activity from dispersed catalytic nanoparticles. Another strategy to improve the overall speed of tubular micromotors is the application of carbon nanomaterials to obtain a rough catalytic inner layer [67,68]. Microtubes prepared by electrodeposited methods possess higher surface roughness, and are superior to conventional microtubes prepared by the rolled-up technique. The underlying mechanism is that a rougher surface promotes bubble generation by enhancing bubble nucleation [69,70]. The first description of an electrodeposited microtube reported a very high speed, i.e., more than 3000 μm/s in 10% H_2_O_2_ solution [71]. They can move at a speed of ~25 μm/s in an extremely low fuel concentration of 0.2% [72]. Large, tubular micromotors fabricated by a template-assisted approach show a fast speed of 183 μm/s in a low concentration H_2_O_2_ of 5% [73].

## 3. Bioactive Fluid as Fuel

### 3.1. Water

The commonly-used catalytic fuel, hydrogen peroxide, hinders practical applications in vivo. The micromotors which utilize water to produce bubbles extend the choices of fuel. Water, the fluids of most living organisms, could be applied to generate hydrogen or oxygen to propel micromotors through reactions between water and metal or photocatalytic water-splitting reaction [74,75]. However, most photocatalytic reactions cannot efficiently generate abundant bubbles to enable the autonomous motion of micromotors. Some kinds of active metals, like potassium, calcium, and sodium, are too violent in their water-splitting reactions to be contained in the design of micromotors; however, magnesium [76] and aluminum (Al) are rather stable due to the formation of a passivation layer on the surface [77]. Consequently, the problem of the passivation layer must be carefully dealt with to make both metals reactive. Gao et al. [78] first reported a water-driven micromotor with a remarkable speed of 3000 μm/s and a very large driven force of 500 pN in water. Both the speed and the driven force are much larger than previously reported common catalytic Janus particles [51], while in other environments, the micromotors also maintained a very fast speed ~500 μm/s. They used aluminum to reduce water to hydrogen. An aluminum alloy, Al-Ga, is utilized to address the passivation layer formed on the Al surface. Al-Ga/Ti microspheres were partially coated on the hemisphere to generate bubbles via chemical reactions. Bubbles were ejected from the Al-Ga hemisphere side to provide a powerful directional driving thrust. The superfast speed of the water-driven Janus motor was partly due to the rather large size (a diameter of 10 μm) of the generated bubbles, and the excellent mobility was apparently related to the larger motor size (average diameter of 20 μm), which means larger bubble size and larger catalytic surface area. While the reactions between Na, Ca, and water are too extensive to control, Mg has become rather attractive due to its moderate reaction rate with water, and its biocompability [67]. Wu et al. [79] constructed an red blood cell (RBC) membrane-coated Mg (RBC-Mg) Janus micromotor mimicking natural motile cells which are powered solely by water. One side of the Mg particles is selectively coated by RBC membranes to asymmetrically generate hydrogen bubbles to endow micromotors with an average speed of 172 μm/s.

Mou et al. [80] reported a Mg-based Janus motor, the majority of which was coated by Pt microspheres, exposing a Mg core. They used an aqueous solution of sodium bicarbonate (NaHCO_3_) to dissolve the Mg(OH)_2_ passivation layer and gained a speed of 75.7 μm/s. The same group further demonstrated the autonomous motion of Mg/Pt Janus micromotors in simulated body fluids (SBF), in which a thermosensitive poly(N-isopropylacrylamide) (PNIPAM) layer is included to fulfill temperature-dependent control [81]. The Mg(OH)_2_ passivation layer is removed by pit corrosion of chloride anions and the buffering effect of SBF. Bubble ejection propels the motor forward with a fast speed of 95 μm/s to yield efficient drug delivery. Wang’s group also built another water-driven, light-activated TiO_2_/Au/Mg micromotor which demonstrates a speed of 110 μm/s [82]. Compared to other peroxide-driven micromotors used in pollutant cleaning, the water-driven ones offer complete and rapid clean up, without the use of a peroxide fuel or a decontamination agent. The micromotor was designed with a small opening to expose the Mg core to surrounding water to aid the directional hydrogen ejection. The motors adopted a similar microsphere size to that in reference [78], yet displayed a much lower speed. The reason may be that the small opening results in a small bubble size and catalytic surface area. Water-powered chitosan-based Janus micromotors with a similar structure, i.e., a small opening, were used to kill bacteria [83]. The micromotor demonstrates a speed of 72.6 μm/s in seawater, offering a 27-fold improvement in antibacterial efficiency compared to the static clearance of chitosan-coated microparticles.

### 3.2. Native Acid

There exists a native acid environment in vivo which provides an ideal biocompatible choice for fuel. Native acids are found in cellular by-products and the gastric area [84,85]. Carbonate-based materials have been widely used in the biomedical area due to their properties of biodegradation and nontoxicity [86,87]. They present a self-propelled biocompatible carrier for drug delivery in acid [88]. They are also applied as carriers to load biologically-active compounds [89]. CaCO_3_ particles are mixed with tranexamic acid to generate bubbles, and they rapidly navigate through aqueous solutions at a velocity of up to 1.5 cm/s [90]. These motors act effectively as a hemostatic agent, and halt severe hemorrhage in vivo. Tumor tissues induce acidosis and generate lactic acid in anaerobic glucose metabolism [91,92]. A faintly acidic environment with average pH 6.5 and a lower pH 5.0–5.5 is observed in cellular components [93]. It has been confirmed that carbonate motors move in the extremely weak acid generated by tumor cells in the absence of external surfactants and fuel [52]. CaCO_3_ Janus particle motors were observed to display slower, but effective motion. 

**Figure 4:**
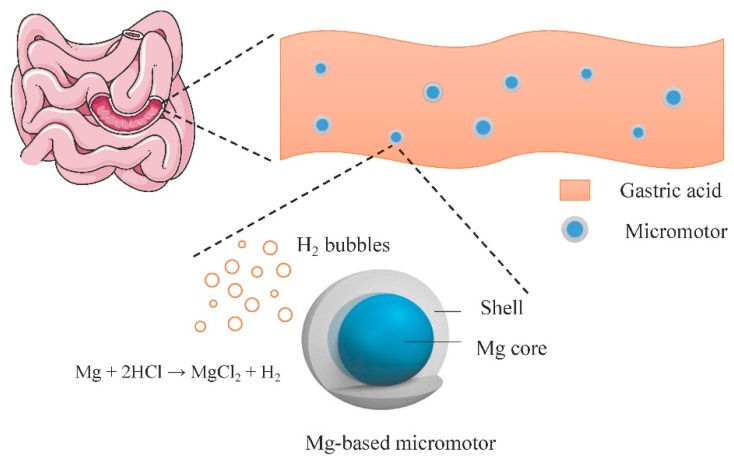
Micromotor body generates hydrogen bubbles to propel the micromotors in the gastric tract.

The gastric acid fluid has been a natural choice as a realistic solution for micromotor fuel [36]. Moreover, Mg/Zn-based micromotors hold great promise for in vivo use, especially for drug delivery in the gastric tract, due to their unique properties, including acid-driven propulsion, the autonomous release of payloads, as well as nontoxic self-destruction. The dissolvable metal body of the micromotors demonstrates efficient motion in harsh acidic conditions and in the absence of external fuel at high speeds. The dissolution of the Zn or Mg-based motor body generates hydrogen bubbles to propel the micromotors ahead in the gastric tract, as illustrated in Figure 4. Gao et al. presented the first study of the use of bubble-driven tubular motors in vivo using a live mouse model [94]. Displacement of the Zn micromotors in gastric acid resulted in the generation of hydrogen bubbles to propel the micromotors at a high speed of 60 μm/s. The motors show dramatically improved retention of payloads in the stomach in comparison with common, orally-administrated payloads. With the motor body dissolving in the gastric fluid, the payloads were gradually released without creating toxic residues. The feasibility of Mg-based micromotors has been confirmed [75]. Authors also reported Mg-based micromotors which are coated with an enteric polymer layer which is dissolved to activate the propulsion at the target site [37]. Authors present the first in vivo drug-delivery application of bubble-driven micromotors which carry clarithromycin to treat gastric bacterial infection in a mouse model [12]. The micromotor consists of a TiO_2_ shell and Mg core which generates hydrogen in gastric media to propel ahead with an average speed of ~120 μm/s. The motor displays effective drug delivery and intensive bacteria clearance in vivo, in comparison with passive delivery manner; no apparent toxicity was observed. Tubular micromotors powered by water also display desirable performance. Recently, authors reported a Mg-based tubular micromotor with polymer coating capable of precise positioning and retention in the gastrointestinal tract with an average speed of 60 μm/s [37].

## 4. Enzyme-Driven Micromotors

### 4.1. Enzyme as a Fuel Source

Enzymes are biomolecular catalysts which accelerate chemical reactions and induce most biological motion at the cellular level. There are mainly three types of molecular motors in the cells: myosins, kinesins, and dyneins. Myosins move along actin filaments, while the latter two move along microtubules. They could not only power the motion of biological structures, but were also capable of providing driving force to enable the motion of synthetic structures. Enzymes with good biocompatibility are an ideal choice to minimize from the toxicity of fuel solution.

Exposed to oxygen, catalase is a common enzyme found in almost all living organisms, like bacteria, plants, and animals. It aids in the decomposition of hydrogen peroxide to water and oxygen. The glucose oxidase enzyme (GOx), also called *notatin*, is an oxido-reductase that catalyses the oxidation of glucose to hydrogen peroxide and other components. Ureases refers to an enzyme that catalyzes the decomposition of urea into carbon dioxide which is applied in motor propulsion.

Similar to the case of Pt-catalyzed motors, enzymes could be conveniently placed on the hemisphere of a Janus particle. Sometimes, these enzymes are encapsulated into cavities of motors to prevent degradation in the presence of proteases. Besides catalase, urease could also be used to functionalize microparticles to promote directed chemotactic movement up the substrate gradient [95]. Moreover, the diffusion of the motor that couples glucose oxidase and Pt-coated nanoparticles is significantly enhanced in the absence of H_2_O_2_ [96].

### 4.2. Enzyme-Powered Micromotors

Catalase has been applied as an alternative to platinum to propel metallic nanorods [14]. There are also successful applications of urease [95,97] or GOx [98] in powering Janus motors. Schattling et al. [99] coat one hemisphere with an enzyme pair GOx and catalase, which exhibit enhanced diffusion behaviors. However, the combustion products of both urease and glucose oxidase are H_2_O_2_ and ammonia, which are considered somewhat cytotoxic. As an effect to deal with the problem, authors have taken one step forward to employ the tandem reaction between GOx and catalase [99,100]. During the reaction, the generated H_2_O_2_ was converted by calatase into oxygen and water. Dual-enzyme catalase and urease incorporated polystyrene particles also demonstrated enhanced diffusion due to thermal effects [97]. A self-electrophoresis mechanism could even drive five-enzyme conjugated nanorods [101]. Although phroresis-driven micromotors successfully proved the feasibility in enzyme-enabled motion, random motion due to Brownian fluctuation makes it difficult to meet the requirement of realistic applications.

**Figure 5:**
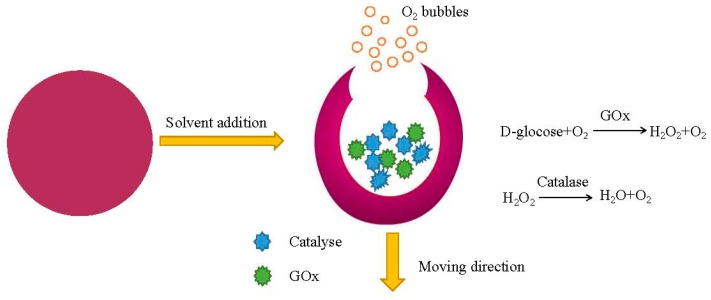
Supramolecular structure of the enzyme-driven stomatocyte micromotors. The motor is driven by a combination of catalyse and glucose oxidase.

The authors constructed Janus nanomotors based on hollow mesoporous nanoparticles, which are powered by three kinds of enzymes: catalase, urease, and GOx [98]. The same group also constructed catalase-coated mesoporous Janus nano-motors which were as small as 90 nm [102]. Simmchen et al. [103] described an asymmetrically-functionalized nanomotor by immobilizing catalase on one side of the particle. The asymmetrical production of bubbles gives increases to create driving force to power the motion of the whole system. These nanomotors generate a force of ~64 fN, which is comparable to the propulsion force provided by a chemically-driven nanomotor [98]. Abdelmohsen et al. [100] reported self-propelled supramolecular stomatocytes in which enzymes catalase or both catalase and glucose is placed inside to generate bubbles, as shown in Figure 5. The structure acquires a propulsion force by ejecting bubbles from a very small pore. The enzyme-powered nanomotor shows a maximum speed of 60 μm/s, which is 3 times higher than that of platinum-driven stomatocytes nanomotors designed by the same group [104].

From the view of physics, enzyme-driven tubular micromotors show better locomotion performance than spherical ones. Thus, researchers have loaded enzymes onto the inner surfaces of tubular motors. Sanchez et al. firstly reported an enzyme-powered tubular micromotor which exhibits ultrafast speed. Tubular motors in which catalase enzyme was contained were able to move at a high speed of 226.1 μm/s at a very low H_2_O_2_ solution [72]. The authors later immobilized the enzyme catalase into the conical micromotor to obtain an enzyme-powered tubular motor in the presence of aquatic pollutants with 2% peroxide [105]. A similar design of catalase-powered micromotor with GO_X_ could be used to decompose sarin stimulant diethyl chlorophosphate (DCP) [106]. Motors consisting of a natural tissue radish body and a combination of catalase and peroxidase were constructed, and they demonstrated an enhanced ultrafast speed of 5000–28,000 μm/s in dilute peroxide solution [107]. Tubular motors incorporated by heat-sensitive gelatin containing gold nanoparticles, doxorubicin, and catalase could rapidly deliver the doxorubicin to targeted cancer cells with a speed of 68 μm/s [108]. The gelatin hydrogel undergoes phase transition to release the loaded doxorubicin under near infrared ray (NIR) irradiation.

## 5. Conclusions and Perspectives

Self-propelled micromotor could act as a perfect tool in various fields. However, the low motility and fuel toxicity hamper their practical application. In this paper, the latest progress in constructing fast and biocompatible, self-propelled micromotors, driven by low-concentration fuel, water, and enzymes were reviewed. These motors have obtained good biocompatibility and motility due to the use of nontoxic fuel, and the recoil forces provided by bubbles. A representative review of the methods and speed of fast bubble-driven micromotors in the biocompatible fuel can be found in Table 1.

In the case of low-concentration fuels, the motion performance of spherical motors could be enhanced by enlarging particle sizes and selecting hollow structures of shell and capsule. Tubular micromotors demonstrate better motion performance than spherical ones due to their 1D manner of the bubble bursting. Moreover, tubular micromotors could acquire faster speeds by optimizing the geometry, namely, by reducing the ratio of length to the radius or enlarging semi-cone angle. To maintain a fast speed, motion efficiency must be promoted in the case of reducing fuel concentration. For instance, the authors often fulfill the task by enlarging the reaction area. A review of the methods to complement reduced concentrations of toxic fuels for micromotors is presented in the “Methods” of Table 1.

The most important thing in water-powered micromotors is the material used to generate bubbles, which is mostly Mg and Al. Mg-incorporated Janus particles with tubular micromotors show good motility. A passivation layer of Mg(OH)_2_ formed in the reactions hampers the chemical reaction. Precise methods must be applied to handle the passivation layer formed on the metal surface. Native acid provides an ideal platform for micromotors which do not need external chemical fuel. As an alternative to platinum, enzymes themselves could propel micromotors in dilute peroxide or without peroxide completely. This technique could be implemented by combining several kinds of enzymes, like catalase and glucose or catalase, urease, and glucose oxidase.

## Figures and Tables

**Table 1:** A representative review of preparation methods and speed of fast bubble-driven micromotors in biocompatible fuel. An absolute speed is described as the criteria for fast micromotors. A concentration of 5% is regarded as the criteria for “Low concentration”. These motors reach a fast speed (>1 bl/s), and even a superfast speed (>10 bl/s), with an ingenious design. The unit of relative speed bl/s refers to body length/second.

Type	Specific Type	Methods	Speeds (μm/s/bl/s), Diameter/Length (μm), Fuel Concentrations
Low concentration of peroxide	Janus micromotors	Slowing down the rotational diffusion Microporous large carbon motors Nanoshell motors multilayer hollow capsules	140,000/3111, 45, 5% [33] 190/3.17, 60, 2% [55] 25/1.25, 20, 0.1% [56] 20/4.22, 4.74, 0.001% [57] 140/17.5, 8, 3% [60]
	Tubular micromotors	Increased solution temperature Hierarchical nanoporous walls Atomic layer deposition Embedding nanoparticles Enlarging rougher surface	>400/4, 100, 5% [21] 100–1000/6.45–38.76, 15.5–25.8, 5% [32] 10,000/200, 50, 5% [20] 120/6, 20, 0.2% [62] 1000/20, 50, 5% [63] 183/1.22, 150, 5% [73]
Bioactive fluid	Water	Al-Ga Janus micromotors RBC-Mg Janus micromotors Pt microspheres exposing a Mg core Light-activated TiO_2_/Au/Mg micromotor	3000/150, 20 [71] 172/8.6, 20 [109] 75.7/3.785, 20 [80] 72.6/3.63, 20 [83]
	Native acid	Carbonate-based materials Zn/Mg-based motor	15,000/1500, 10 [90] 60/4, 15 [37] 120/6, 20 [12]
Enzyme-driven micromotors		Catalase Glucose oxidase Catalase and peroxidase Supramolecular stomatocytes	60/182, 0.33, 111 mM [100] 226.1/10, 22.6, 1.5% [72] 5000–28,000/0.71–4, 7000, <1% [107] 59/2.95, 20, 2% [108]
